# Development and Validation of the Acceptance of Violence Against Women Scale (AVAWS)

**DOI:** 10.1186/s41155-025-00351-4

**Published:** 2025-06-19

**Authors:** Tamyres Tomaz Paiva, Cicero Roberto Pereira, Estela Mírian Lima da Silva, Carlos Eduardo Pimentel

**Affiliations:** 1 Faculdade de Enfermagem Nova Esperança (FACENE), João Pessoa, Brazil; 2 Federal University of Paraiba, João Pessoa, Paraiba Brazil

**Keywords:** Intimate partner violence, Cultural sexism, Gender prejudice, Discrimination, Aggression

## Abstract

**Background:**

Violence against women is one of the most dramatic expressions of subjugation to which women are subjected in all patriarchal societies. The present research comprised four studies to develop and validate the Acceptance of Violence Against Women Scale (AVAWS).

**Methods:**

Participants in these studies included 15 experts (Study 1), 305 general respondents (Study 2), 293 respondents (Study 3), and 300 respondents (Study 4).

**Results:**

In Study 1, we provided evidence of content validity for a theoretically appropriate, accurate, clear, and relevant set of items and scenarios to be included in the AVAWS. In Study 2, we analyzed the factor structure of the scale and identified a multidimensional measure representing five types of violence (physical, sexual, psychological, moral, and economic). Study 3 confirmed this factor structure and showed that the AVAWS is best represented by a bifactor model that assesses both general (G-factor) and specific (S-factors) support for violence against women. Finally, in Study 4, we experimentally demonstrated the criterion validity of the AVAWS and showed that it is sensitive to manipulations of cultural sexism.

**Conclusion:**

It is concluded that the AVAWS is a valid and reliable instrument for assessing social support for violence against women, being a pioneer in documenting cultural sexism as an organizing principle of this support, with potential applications in clinical, educational, and legal contexts.

**Supplementary Information:**

The online version contains supplementary material available at 10.1186/s41155-025-00351-4.

## Introduction

In contemporary patriarchal societies, violence against women remains a pervasive problem that reflects deeply rooted gender inequalities and archaic social norms (Barker, 2016; Sikweyiya et al., 2020; Tonsing & Tonsing, 2019). Historically, women have been relegated to subordinate roles limited to the domestic sphere, while men have occupied positions of public importance and authority (Pratto & Walker, 2004; Walker, 1989). Despite advances in women's rights in Western democratic societies, women continue to be exposed to various forms of violence due to the ongoing influence of cultural and social gender ideologies.

The violence against women is multifaceted and encompasses a full spectrum of gender-based assaults (Jordan et al., 2010). In heterosexual relationships, violence often arises from a distorted understanding of male dominance in which women are viewed as property acquired through marriage, resulting in dehumanization by their male partners (Muehlenhard & Kimes, 1999; Rudman & Mescher, 2012). This violence transcends physical boundaries and invades psychological, sexual, economic, and moral domains, causing immense harm and suffering (Ali et al., 2016; Jordan et al., 2010; WHO, 2020). Physical violence inflicts bodily harm, sometimes with fatal consequences (Jordan et al., 2010). Psychological violence, which aims to control and belittle, undermines self-esteem and emotional well-being (Ali et al., 2016; Jordan et al., 2010). Sexual violence violates intimacy and forces women into unwanted relationships through intimidation or violence (Jordan et al., 2010). Economic violence limits financial independence and impairs women's autonomy (Adams & Beeble, 2019; Ali et al., 2016). Moral violence tarnishes reputations through slander and insult (Ali et al., 2016). Despite the severity of these forms of violence, Brazil has made significant progress through the implementation of important laws. The first major milestone was Law 11.340 (2006), known as the"Maria da Penha"law, which strengthened women's rights by providing better legal protection, promoting awareness campaigns and offering psychological support to victims. More recently, Law 13.104 (2015) was enacted, which classifies femicide as a heinous crime and provides for more severe penalties of 12 to 30 years imprisonment. While these legal advances represent significant progress, challenges remain in fully enforcing these protections and addressing gaps in support for women. Today, violence against women is recognized as a serious public health problem and a punishable crime in many Western democracies (WHO, 2013).

However, there are still alarming statistics that point to the prevalence of this violence. A World Health Organization study of 80 countries found that 35% of women worldwide have experienced physical or sexual violence by an intimate partner and that these partners are responsible for 38% of homicides against women worldwide (WHO, 2013). These disturbing numbers underscore a troubling reality: violence against women continues to be a deeply entrenched descriptive norm that is shared by society. Consequently, understanding this societal support is critical.

This study addresses this critical issue by developing a comprehensive measure of societal support for various forms of domestic violence against women. By shedding light on the factors that perpetuate this violence, we aim to pave the way for targeted interventions and social change, and to advocate for a world where women are free from the shackles of violence and inequality.

### Measure of the acceptance of violence against women

Various instruments have been employed worldwide to assess the social acceptance of violence against women, with studies spanning countries such as Germany, the United States, France, Spain, and Brazil. These tools often focus on the attribution of responsibility in cases of sexual violence, particularly through constructs like Rape Myth Acceptance (Burt, 1980; Gerger et al., 2007; Peters, 2006; Scarpati et al., 2014). A prominent example is the Acceptance of Modern Myths about Sexual Aggression Scale (AMMSA), developed and validated in Germany by Gerger et al. (2007). Other instruments, such as the Domestic Violence Myth Acceptance Scale (DVMAS; Peters, 2006), capture multiple dimensions, including exoneration of the perpetrator, victim-blaming, and the trivialization of violence. Lelaurain et al. (2018) found that the DVMAS reflects a general tendency to accept violence, underscoring the pervasive nature of these beliefs.

Other instruments address positive attitudes toward domestic violence. The Intimate Partner Violence Attitude Scale in Dating Relationships (Fincham et al., 2008) measures attitudes using a three-factor structure: abuse, control, and violence. Similarly, the Acceptability of Intimate Partner Violence Against Women Scale (A-IPVAW), developed and validated by Martín-Fernández et al. (2018), assesses a one-factor structure that includes support for physical, coercive, and verbal violence, as well as controlling behaviors and emotional violence.

While these instruments provide valuable insights, many do not fully encompass the theoretical complexity of various forms of violence. For example, the Acceptance of Myths about Intimate Partner Violence Against Women (AMIVAW) scale (Mégias et al., 2018) assesses beliefs about violence through items like:"A man who abuses his partner does so because he doesn’t know how to behave otherwise."Similarly, the Intimate Violence Responsibility Scale (I-VRS; Yun & Vonk, 2011) addresses cultural beliefs, as seen in the item:"It is part of my culture to treat my partner that way."Other widely used tools, such as the Acceptance of Couple Violence Scale (ACVS) (Foshee et al., 1998), which was validated in Brazil by Pimentel et al. (2017) as the Acceptance of Dating Violence Scale, include items like:"A girl who purposely makes her boyfriend jealous deserves to be hit."This scale reflects beliefs regarding the reciprocal nature of partner violence. Additionally, the Spousal Physical Violence Scale and the Sexual Assault Scale, developed by Nayak et al. (2003) and validated in Brazil by Nascimento (2015), measure attitudes toward physical and sexual violence. Examples of their items include:"Many women falsely report rape for attention"and"A woman’s nagging is a major cause of violence in the home."Each of these tools offers distinct definitions and assessments of violence, based on different theoretical approaches, highlighting the varied dimensions and nuances in understanding violence against women.

Despite the usefulness of the existing tools, a crucial gap remains. None of these tools encompass the different forms of violence that women experience within the complex dynamics of abusive relationships. Knowing that gender inequalities underlie discrimination in power dynamics, we recognize the need to explore the multifaceted domains in which these relationships occur. To address this limitation, our study pioneers the development and validation of the Acceptance of Violence Against Women Scale (AVAWS). This innovative instrument incorporates realistic scenarios with fictional characters representing victims and aggressors in everyday relationship situations to provide a nuanced understanding of societal attitudes toward various forms of violence against women. By grounding our assessment in real-world contexts, we seek to minimize the risk of oversimplified conclusions and provide a more nuanced and comprehensive perspective in psychological studies focused on gender dynamics (Cruz & Irffi, 2019; Machado et al., 2020).

### Overview of studies

We present the Acceptance of Violence Against Women Scale (AVAWS), developed to measure societal support for violence against women through a comprehensive four-part research program. The AVAWS uses real-world scenarios of violence from media reports accessible on social media. Our main hypothesis is that societal support for various forms of violence against women condenses into a single factor that represents people's general propensity to endorse such violence. Based on Law 11.340 (2006), the factor structure includes five specific types of violence: physical, psychological, economic, moral, and sexual.

The development and validation of the Acceptance of Violence Against Women Scale (AVAWS) in the Brazilian context is essential due to the high rates of violence against women that the country faces. By presenting alarming statistics — such as the fact that, on average, a woman is physically assaulted every 17 min (Mapa da Violência Contra a Mulher, 2018) — we highlight the urgent need for research that assesses societal attitudes towards such violence. The fact that Brazil ranks fifth in the world with a murder rate of 4.8 murders per 100,000 women (Waiselfisz, 2015) underscores the severity of the problem and highlights the importance of a deeper understanding of public perceptions of violence against women. These statistics are not just numbers; they reflect a critical reality that positions Brazil as a relevant social context for examining societal attitudes towards violence against women. The shocking figure of approximately 15,925 women who were victims of femicide in 2017, with 95% of these deaths committed by intimate partners (Mapa da Violência Contra a Mulher, 2018), highlights the intimate nature of this violence and the societal dynamics at play. By placing the development of AVAWS in this context, we ensure that the scale is not only relevant, but also able to capture the nuances of societal attitudes shaped by real and pressing concerns about violence against women in Brazil.

In sum, the integration of these statistics supports the contention that Brazil provides a critical social backdrop for assessing societal attitudes about violence against women, making the findings of this research both timely and necessary. Our research was conducted in four different studies. In Study 1, we carefully elaborated the items of the scale and then assessed their content validity. In Study 2, we examined the factor structure of the scale, while in Study 3, we conducted a confirmatory factor analysis to examine the fit of the proposed measurement model while testing the convergent-discriminant validity of the measure. Study 4 deepened the investigation by conducting a random group experiment to examine the sensitivity of AVAWS to contextual variation regarding a theoretical history of violence against women.

### Study 1. Item development and content validity

In this study, we developed a set of vignettes depicting violence, about which items would be presented to measure the societal support for various types of domestic violence. We also examined the vignettes and scale items for content validity. In the first step, we developed 10 vignettes representing the five forms of domestic violence that we identified in the literature, with two vignettes specified for each type of violence (see Table 1). These vignettes were based on media stories published online by the leading newspapers in Brazil. We researched news reports to gather real-life examples and selected those that best illustrate instances of violence that are often not recognized as such, but rather perceived as acts of jealousy, care, protection, or the expected duties of a married woman. We summarized the reports as much as possible to highlight only their main characteristics. We were careful to change the characters'names and omit information about the social and geographic environment in which the situations occurred, to anonymize each case.

**Table 1:** Scenarios of domestic violence

1. Maria and Jose are married. A few days ago, Maria was with friends, when Jose arrived and forcefully grabbed her, leaving visible marks on her arm	PHV
2. Diana and Lucas are married. Diana wanted to go to a female friend's birthday party, but Lucas didn't want to go. When Diana was leaving to go to the party, Lucas pulled her so hard that she fell to the ground	PHV
3. Estela and João are married. During a discussion, Estela said that she wanted a separation, and João responded,"If you won’t be mine, then nobody will have you"	PV
4. Emily and Marcos are married. Marcos said that if Emily didn't do what he wanted, then he would leave her. Marcos also said that she would not find any other person who loved her as much as he loved her	PV
5. Felipe and Clara are married. Clara complains that she is unable to perform sexual acts the way that Felipe demands of her	SV
6. Carlos and Ana are married. Ana complains that every night that they are in bed, Carlos demands that she sexually stimulate him so that he gets enjoyment from it, even when she is not in the mood	SV
7. Pamela and Diego are married. Pamela says that Diego makes it difficult for her to work. Diego states that his income is enough to maintain the home	EV
8. Estela and Gustavo are married. Gustavo saw some messages on Estela's mobile phone, and afterward he broke the phone by throwing it against the wall	EV
9. Flora and Geraldo are married. Geraldo says to his friends that Flora doesn't behave like a married woman	MV
10. Silvana and Guilherme are married. Guilherme says that Silvana doesn't behave like a real housewife	MV

Legend: *PHV* physical violence, *PV* psychological violence, *SV* sexual violence, *EV* Economic violence, *MV* moral violence

In the second phase, we developed four items for each vignette. Two of the items reflected the acceptance of violence, as demonstrated by previous research within the framework of the Justified Discrimination Model (JDM; Paiva & Pereira, 2021; Pereira et al., 2018): “What he [male character] did is understandable”; and “[Male character] merely acted as any husband would.” The other two items represented a rejection of violence: “[Male character] should have kept quiet”, meaning not to shout or yell or otherwise be verbally abusive in the Brazilian context; and “[Male character]’s behavior was unjustified.” After developing the violence vignettes, we moved on to the third phase. We performed content validity analysis to evaluate the theoretical reliability and credibility of the vignettes and proposed items. Content validity involves two crucial aspects: a) knowing whether the writing of the items is understandable by members of the target population; b) assessing the theoretical adequacy of the content of the items written to measure the construct (Boateng et al., 2018). We assessed these two aspects following the recommendations of Boateng et al. (2018), conducted cognitive interviews to evaluate items’ intelligibility by some individuals from the target population, and examined the theoretical relevance of each item to measure violence against women according to some expert researchers in the topic.

### Study 1a. Expert Review

#### Method

*Participants*. Expert raters were invited by email, which included a form explaining the aims of the study and a request to collaborate in the theoretical analysis of the items. We selected raters through a rigorous analysis of their Lattes Curriculum, focusing on their expertise in studying discriminatory attitudes and behaviors toward social minorities. Ten raters were approached, but only five of them considered themselves knowledgeable enough to participate in a study on specific issues of violence against women. They were psychologists aged 24–33 years (*M* = 28.60, *SD* = 3.36; 4 women and 1 man) and were working on actions to prevent intimate partner and dating violence at the time of the study.

*Instrument*. We presented the instrument to the raters and asked them to analyze each item in relation to the following four aspects: 1) domain/adequacy: whether each item adequately represents the presented scenarios of violence (physical, psychological, sexual, economic and moral); 2) accuracy: whether each item adequately operationalizes the construct; 3) clarity: whether the items can be understood by all levels of the target population; and 4) relevance: whether the items are psychologically relevant to describe the construct in question (Dimitrov, 2012). The items were evaluated on a scale from 0 to 10, in which 0 represents complete inadequacy and 10 represents complete adequacy in all the aspects evaluated.

*Procedures*. The instrument was distributed online via an e-mail with a link to access the instrument. For the domain/adequacy of the items, Cohen’s coefficient kappa (k) was calculated to evaluate the raters’ agreement about the vignettes and each rater’s judgment regarding each item’s agreement with the pre-established dimensions. The content validity coefficient (CVC) proposed by Hernández-Nieto (2002), which evaluates inter-rater agreement, was also calculated with regard to each item’s accuracy, clarity and relevance to the scenario (Dimitrov, 2012).

*Data analysis.* Content Validity Coefficient (CVC; (Hernandez-Nieto, 2002) was used to estimate the content validity parameter. The cutoff criterion was 0.70 or higher for all CVC estimates (Hernández-Nieto, 2002). Cohen’s coefficient kappa (k) and intraclass correlation coefficient (ICC) were used to examine reproducibility. Data was analyzed in the SPSS (v. 24.0).

## Results

The results indicated an absolute intraclass correlation coefficient (ICC) of 0.84 for rater agreement and a kappa of 0.97 for the adequacy of the items regarding the vignettes, denoting excellent agreement. This analysis indicated both the theoretical consistency of the instrument and the degree of consensus among the raters (Bland & Altman, 1986; Cicchetti & Sparrow, 1981). The raters agreed on the accuracy (CVC = 0.96), clarity (CVC = 0.93), and relevance (CVC = 0.95) of the items. Additionally, a total scale-level total content validity coefficient (S-CVCt) of 0.95 for all criteria was obtained for all items. According to Hernández-Nieto (2002), a threshold of CVC ≥ 0.80 can be considered satisfactory. In other words, the items were accurate, clear and relevant to measure the construct in question. The raters stated that only scenario 2 (physical violence) (see Table 1) was not sufficiently clear to remain in the later stages.

### Study 1b. Cognitive Interviews

#### Method

*Participants.* Ten university students participated in this analysis. The participants'ages ranged from 19 to 24 years (*M* = 21.60, *SD* = 1.43); the majority were female (70%) and were between the 3rd and 9 th semester of undergraduate studies. Approximately 80% of the participants stated they had witnessed some type of violence against women.

*Instrument*. We asked the participants to answer, on a scale from 1 to 4 (1 = not at all, 4 = totally), how much they perceived each item as being written in a clearly understandable manner. We used two vignettes each for sexual, moral, economic and psychological violence, and only one vignette for physical violence as a result of the raters’ analysis. Thus, a total of nine vignettes were presented in the evaluation. We calculated the mean agreement of the clarity criterion for the items in each vignette to assess the extent to which the questions reflect the domain of interest, and the answers yield valid readings because they are grammatically correct and understandable (Boateng et al., 2018; DeVellis, 2012).

*Procedures and data analysis.* During recruitment, researchers approached students and obtained their informed consent to participate. The study was conducted in person in a classroom following a class period. To ensure clarity of the questionnaires, participants were first asked to read each item aloud. Then, the researchers discussed with the participants to verify that they understood the content of each question. After confirming the comprehensibility of all items, participants were asked to rate the extent to which they felt each item was clearly understood. To assess item comprehensibility, a comparison was made between the item comprehension mean and the midpoint of the response scale (i.e., 2.5). This comparison was made using a *t*-test. The data collected during this process were analyzed using SPSS software (version 24.0). This careful procedure ensured that the participants fully understood the items of the questionnaire, thus increasing the reliability and validity of the results of the study.

### Ethical aspects

All studies were approved by a research ethics committee that adhered to the guidelines of the Brazilian National Health Council ( Ethics Committee for Research Involving Human Beings, Committee Report: 20076919.2.0000.5188; Ethical Opinion nº 3.624.434) . Participants, who were fully informed of the objectives of the study and the researcher's contact information, voluntarily agreed to participate. The datasets resulting from this comprehensive research program are freely available through the Open Science Foundation's repository platform (https://osf.io/5a4g3/?view_only=60a15acff6a54f0ca7c5947f99039d95), promoting transparency and the dissemination of knowledge in this important area of research.

## Results

According to the raters, we assessed the extent to which the items reflect the societal support to violence with theoretical accuracy. Results indicated that physical violence scenario 1 had a mean of 3.77. The psychological violence vignettes had mean values of 3.92 and 4.00 for vignettes 3 and 4, respectively; the sexual violence vignettes had mean values of 3.82, and 3.70 for vignettes 5 and 6, respectively; and the means obtained for the economic violence vignettes were 3.75 and 3.82 for vignettes 7 and 8, respectively. Regarding moral violence, vignettes 9 and 10 received respective mean values of 3.80 and 3.70. Additionally, all of the vignettes had a mean value higher than the midpoint of the scale, that is, > 2.5 (ts > 6.60, p < 0.001), indicating that the items would be understandable to the target population.

## Discussion

The results of this study strongly support the content validity of the AVAWS items. Of the ten vignettes originally prepared, nine were found to be appropriate based on careful analysis by raters and cognitive interviews. This rigorous process facilitated the selection of the most appropriate vignettes and items for the scale. In particular, Scenario 2 on physical violence, which could not accurately represent the intended latent trait due to its unclear operational definition (according to Dimitrov, 2012), was excluded from subsequent analyzes. Consequently, nine vignettes were retained, each addressing a specific form of violence against women.

The use of interviews was important to determine the target audience's understanding of each scenario and its associated items. This critical step ensured the quality and clarity of the items and increased the robustness of the instrument. These results convincingly demonstrate the content validity of the items and vignettes in the first version of the AVAWS, which was developed specifically to measure societal support for various forms of violence.

It is important to note, however, that although content validity was demonstrated, the study still needed empirical evidence of the factorial validity of the AVAWS. This critical goal was addressed in Study 2, in which rigorous analyzes were conducted to establish the factorial validity of the scale. These subsequent analyzes aimed to further validate the instrument and strengthen its utility as a reliable measure of societal attitudes toward various situations of violence against women.

### Study 2. Exploring the Factor Structure of the AVAWS

In this study, we analyzed the AVAWS factorial validity. We reasoned that individuals'response to the items would reflect their societal support for each type of violence described in Study 1. Thus, we hypothesized that the set of items would load adequately in the respective scenarios, with the collection of vignettes organized into a one-factor structure (i.e., a general latent factor).

## Method

### Participants

A total of 314 undergraduate students participated. Most were working toward degrees in the humanities (42.8%), the standout being psychology (26.1%), and they ranged in age from 18 to 62 years (*M* = 24.77, *SD* = 6.25). They were predominantly female (76.8%), single (84.4%), heterosexual (72.3%), and belonged to the lower middle class (42.4%). Of the respondents, 40.4% were currently dating, and 56.6% had been dating their partner for more than two years.

*Instrument.* The AVAWS comprised nine vignettes of violence against women, with each scenario expressing a specific type of violence (see the vignettes in the online supplementary materials). Each vignette consists of four items. Two of them represent acceptance of violence (e.g., “What [male character] did is understandable” and “[Male character] merely fulfilled his role as a husband”), while the other two items represent the rejection of violence (“[Male character] should have stayed quiet” and “[Male character]’s behaviour is unjustified”). Answers were given respecting a Likert scale that varied from 1 (strongly disagree) to 6 (strongly agree).

*Procedures.* Data were collected online through the Qualtrics platform (Qualtrics, 2014) on various social networks (e.g., Facebook, Instagram, email). We sent the invitation to groups of university students available on these social networks and asked them to access the link to the study and follow the instructions described there after the participant agreed to the consent form. We randomized the order of presentation of the vignettes to avoid the order effect and minimize fatigue and recency effects.

*Data analysis.* We analyzed the data with Mplus version 6 (Muthén & Muthén, 2010) and IBM SPSS Statistics 21 for descriptive statistics and reliability coefficients. We estimated an exploratory factor analysis (EFA; principal-axis factoring method) by using the weighted least squares mean and variance adjusted (WLSMV) estimator for ordinal data. We then conducted a parallel analysis (Horn, 1965) to ensure the stability of the number of factors.

## Results

We tested whether the four items were unidimensional relative to their corresponding vignettes. To this end, we conducted specific EFAs for each vignette. The results showed that the four items loaded in only one factor (see Table S1 in the online supplementary materials). Because the items measured only one factor for each vignette, we calculated a score for support of the violence expressed in each vignette by aggregating the items and inverting those that had a negative sense. With this procedure, we analyzed the nine vignettes to determine whether the factor structure obtained represented the five types of violence or an alternative configuration. The communalities of the items ranged from *h* < 0.98 (item 1 of psychological violence scenario 1) to *h* > 0.19 (item 3 of psychological violence scenario 3).

According to the Kaiser criterion (Kaiser, 1958), the vignettes had an eigenvalue of 3.48, which accounted for 38% of the variance in a single factorial model, meaning that the eigenvalues were above 1. A parallel analysis (Horn, 1965) further confirmed the unifactorial structure. In this analysis, the vignettes showed factor loadings above 0.50, with the exception of psychological vignette 4, which showed negative loadings for the items"should have kept quiet"and"his behavior is unjustified,"both of which were below 0.20. We therefore decided to use the vignette with the highest Cronbach's alpha as the selection criterion. For the vignettes on sexual violence (5 and 6), we selected vignette 6, which had the higher alpha. For the vignettes on economic violence (7 and 8), we selected vignette 7. Finally, vignette 10 was selected for the vignettes on moral violence (9 and 10). The factors (subscales) showed internal consistency, with Cronbach's alpha between 0.56 (psychological violence, vignette 4) and 0.92 (moral violence, vignette 10), as shown in Table S1.

As the aim of this study was to explore the factor structure and identify the vignettes that best reflected participants'support for violence, we selected the vignettes with the highest factor loadings. Finally, based on internal consistency and factor loadings, we selected the five vignettes that best met these criteria. A subsequent factor analysis using only these five vignettes resulted in a single factor with satisfactory internal consistency (see Table 2).

**Table 2:** Factor loadings of the vignettes

Violence scenario	Factor loading (h^2^)
1. Physical	.68 (.47)
4. Psychological	.71 (.50)
6. Sexual	.74 (.55)
7. Economic	.72 (.51)
10. Moral	.77 (.59)
Eigenvalues	2.64
Variance (%)	52.93
Total α	.76

## Discussion

The results of the study are closely aligned with our original hypothesis that the five selected vignettes would effectively measure participants'attitudes toward violence against women. In particular, the exploratory analyzes of the vignettes from Study 1 allowed us to determine the top five vignettes for inclusion in the AVAWS. In addition, our analysis revealed strong internal consistency across most vignettes, with only the psychological violence scenario falling slightly below the expected threshold > 0.61(Landis & Koch, 1977).

Both Study 1 and Study 2 made important contributions by demonstrating the content and factorial validity of the AVAWS. However, one crucial question remained unanswered: Would the proposed structure fit the data, and would this fit outperform alternative factor structures? To answer this question and further improve the reliability of the scale, Study 3 was carefully designed. In this phase, we aimed to determine the fit of the proposed structure by estimating composite reliability coefficients for all vignettes within the scale. This step allowed us to thoroughly test the internal consistency of the scale and ensure that the AVAWS not only accurately measures societal attitudes, but also provides a robust and reliable framework for understanding the complexity of violence against women.

### Study 3. Confirmatory analysis of the factor structure of the AVAWS

This study aimed to confirm the AVAWS factor structure. In addition to the single factor model found in Study 2, we tested alternative models that could improve understanding of the factor structures of the AVAWS. We hypothesized that the societal support for violence against women could be expressed as a general attitude of support for all types of violence (i.e., a G-factor), and could also be expressed specifically, distinguishing among each type of violence (i.e., S-factors). Thus, the existence would be likely of a bifactor structure (i.e., a general factor independent from the five specific factors) or a hierarchical structure (i.e., a first-order general factor measured by the five specific factors). We compared these two factor structure models with each other, and with single-factor and multi-factor measurement models. We examined the convergent-discriminant validity of the AVAWS for measuring individuals'acceptance of violence against women by testing whether it correlates positively with another well-established scale of violence against women that has been widely used in previous studies (i.e., convergent validity) and whether it has low correlations with other constructs that are expected to be less correlated to acceptance of violence, such as personality traits (i.e., discriminant validity). Specifically, we used the Acceptance of Dating Violence Scale (Foshee et al., 1998) for convergent validity and the Ten-Item Personality Inventory (TIPI; Gosling et al., 2003) for discriminant validity.

## Method

### Participants

A total of 293 undergraduate students took part in the study. Their ages ranged from 18 to 44 years (M = 23.16, SD = 4.61), and 63.8% of the participants were women. Most of them were studying humanities (34.2%), with psychology accounting for the largest proportion (21.2%). Most participants reported being single (89.1%) and heterosexual (71.3%). 50.2% stated that they were not very religious. In addition, 38.6% said they were lower middle class. Of the respondents, 42.3% were currently in a relationship and 48.4% had been in a relationship for more than two years. We conducted sensitivity power analysis by using Webpower (Zhang & Yuan, 2018) to assess the power of estimating parameters in structural equation modeling. Using the observed degrees of freedom (148) and setting an acceptable goodness-of-fit to the data (based on RMSEA = 0.05), our sample size (*N* = 293) had power of 0.99.

*Instruments.* We used the AVAWS version obtained in Study 2. For the proposal of convergent validity, we used the Acceptance of Dating Violence Scale (Foshee et al., 1998) as validated by Pimentel et al. (2017). It has 11 items grouped into the following three factors: general dating violence (e.g., “Violence between people who are dating can improve the relationship”, α = 0.72); male-on-female violence (e.g., “A guy who is angry enough to hit his girlfriend must love her very much”, α = 0.64); and female-on-male violence (e.g., “Guys sometimes deserve to be attacked by their girlfriends”, α = 0.82). The participants stated how much they agreed with each statement (1 = totally disagree; 4 = totally agree).

To assess discriminant validity, we used the Ten-Item Personality Inventory (TIPI; Gosling et al., 2003), specifically the Brazilian adaptation by Pimentel et al. (2014). Previous research has provided empirical evidence supporting the reliability, convergent-discriminant validity, and construct validity of the TIPI for measuring individual differences in personality traits across more than 100 cultures, including Brazil (e.g., Boileau et al., 2024; Carvalho et al., 2011; Nunes et al., 2018; Paiva et al., 2021; Silva & Pereira, 2022). It consists of 10 items distributed into the following five factors (1 = strongly disagree; 7 = strongly agree): Extraversion (e.g., extroverted, enthusiastic; *r* = 0.48, *p* < 0.01); agreeableness (e.g., sympathetic, warm; *r* = 0.14, *p* < 0.01); conscientiousness (e.g., dependable, self-disciplined; *r* = 0.31, *p* < 0.01); emotional stability (e.g., calm, emotionally stable;* r* = 0.49, *p* < 0.01); and openness to experiences (e.g., open to new experiences, complex;* r* = 0.23, *p* < 0.01).

*Procedures and data analysis.* We followed the same procedures of Study 2 for data collection. Mplus version 6 (Muthén & Muthén, 2010) was used to estimate the parameters and obtain goodness-of-fit indices for the model. Confirmatory factor analysis (CFA) was performed using the weighted least squares mean and variance adjusted (WLSMV) estimator for continuous ordinal data. We used the following goodness-of-fit criteria (see Byrne, 2012): chi-square/degrees of freedom: acceptable if < 0.08; comparative fit index (CFI) and Tucker-Lewis index (TLI): comparative indices of the model, with > 0.90 indicating a good fit for both; root mean square error of approximation (RMSEA): < 0.05 is indicative of good fit, but up to 0.08 is acceptable (Byrne, 2012); and the weighted root mean square residual (WRMR), which allows values with a cutoff closer to 1.00 (DiStefano et al., 2017).

## Results

**Fig. 1:**
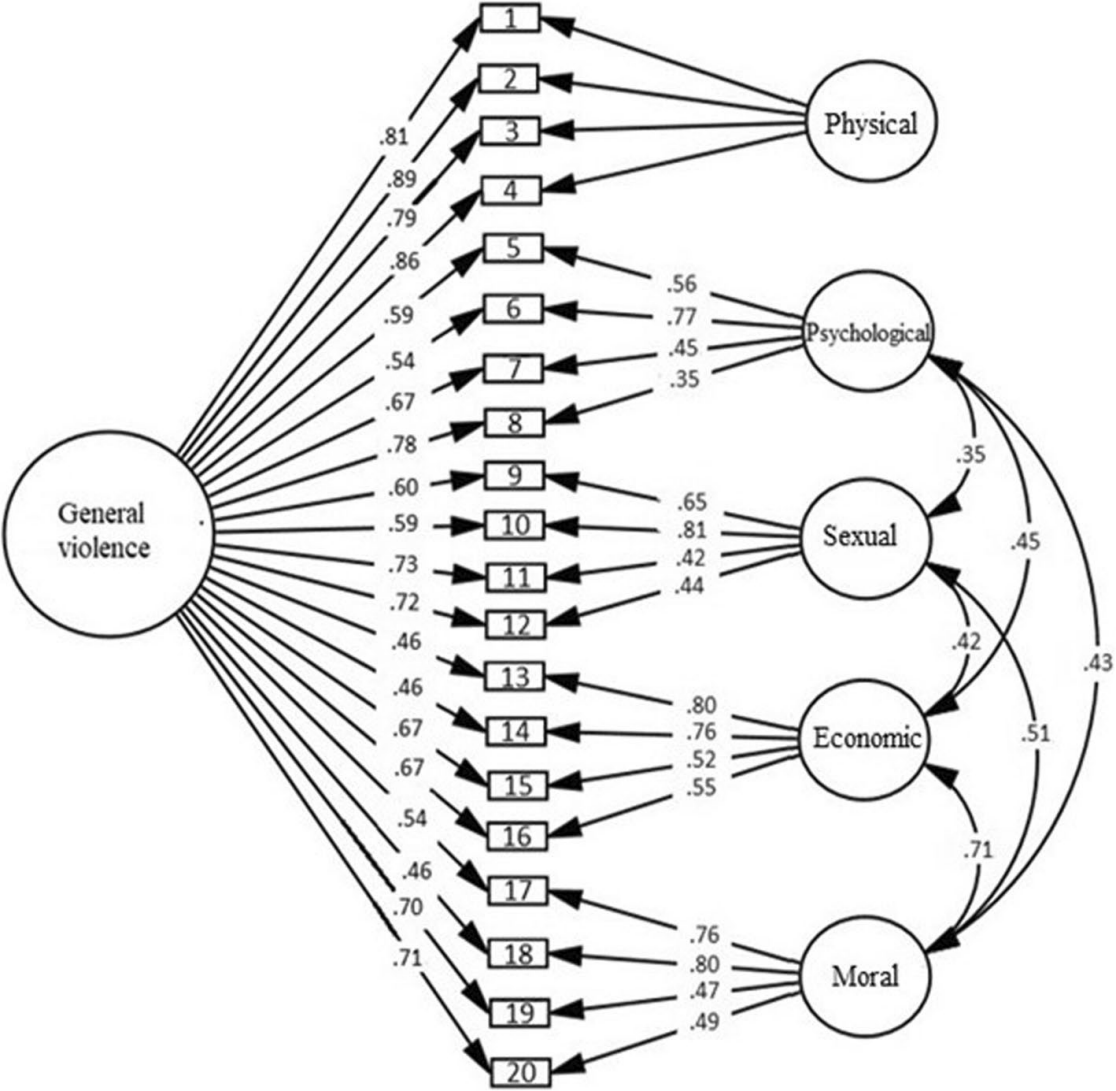
Bifactor structure (S- 1) of the AVAWS

We performed a set of CFAs to address the goodness-of-fit of the following measurement models: a single-factor model; a five-factor model in which the five factors specified each type of violence; a bifactor model consisting of five specific factors and one general factor; and a hierarchical model with five first-order factors and one general second-order factor. Table 3 presents the fit indices of all the models tested. The results showed that the bifactor model (S- 1) fitted the data better than the other models. The estimated factor loadings are shown in Fig. 1.

**Table 3:** Comparison of the measurement models of the AVAWS

Model	χ^2^	Df	χ^2^/df	CFI	TLI	RMSEA (95% CI)	WRMR
One-factor	740.138	170	4.35	.92	0.91	.10 (.09;.11)	1.71
Hierarchical	440.950**	165	2.67	.96	0.95	.07 (.06;.08)	1.21
Five-factor	424.019**	160	2.65	.96	0.95	.07 (.06;.08)	1.14
Bifactor S- 1	400.859**	148	2.70	.96	0.95	.07 (.06;.08)	1.03

*Note:* **p <. 001

We also estimated the composite reliability (CR), which was strong for all the violence domains: physical (0.90), psychological (0.74), sexual (0.82), moral (0.83), and economic (0.85), as well as for the general scale (0.92), which indicates strong reliability (Fornell & Larcker, 1981).

We subsequently explored the convergent-discriminant validity of the AVAWS by estimating its correlation with the Acceptance of Dating Violence Scale and with the Personality Inventory scores (see Table S2). For convergent validity( Campbell & Fiske, 1959), the results showed the following pattern of associations: positive correlations between physical violence and general dating violence, male-on-female violence, and female-on-male violence were observed; psychological violence was positively correlated with general dating violence, male-on-female violence, and female-on-male violence; sexual violence was positively correlated with general dating violence, male-on-female violence, and female-on-male violence; economic violence was positively correlated with general dating violence, male-on-female violence, and female-on-male violence; moral violence was positively correlated with general dating violence, male-on-female violence, and female-on-male violence; and the G-factor of acceptance of violence was positively correlated with general dating violence, male-on-female violence, and female violence. In assessing the discriminant validity (Campbell & Fiske, 1959) of AVAWS in relation to personality traits, only agreeableness exhibited a negative correlation with various forms of violence. This suggests that individuals with lower levels of agreeableness tended to score higher on AVAWS. This finding aligns with prior research indicating that individuals with low agreeableness traits are more predisposed to hostility, antagonism, and lack of cooperation. These traits are intricately linked to the endorsement of violence within intimate relationships (Paiva et al., 2017). Nevertheless, these traits were consistent with the distinction between the constructs, since the coefficients with physical, psychological, sexual, economic, and moral violence and the acceptance of general violence were very low. In sum, these results showed evidence of convergent-discriminant validity of the AVAWS with the acceptance of dating violence and with personality traits.

## Discussion

The results of the study not only confirmed the factor structure of the AVAWS, but also showed a G-factor within the scale. Furthermore, the bifactor structure analysis indicated the existence of different subtypes of violence acceptance, each of which can be studied independently. This indicates the adaptability of AVAWS in measuring general violence (G-factor) and specific types of violence (the S-factors). In addition, the study demonstrated the convergence of the scale with an assessment of the acceptability of intimate partner violence. Given their common focus on measuring violence, AVAWS and the Acceptance of Dating Violence Scale showed moderate correlation, especially for general violence in couple relationships and men's violence against women, whereas the correlation was low for women's violence against men. This result was to be expected given that AVAWS assesses violence against women, while female-on-male violence evaluates the opposite phenomenon. Furthermore, the discriminant validity of AVAWS was clearly demonstrated. This was evident in the results, as AVAWS scores were generally not correlated with personality traits, highlighting the difference between the constructs measured. AVAWS assesses support for violence against women, whereas the TIPI instrument assesses other constructs, particularly personality factors.

To this point, our studies have provided robust evidence of AVAWS's factorial, convergent, and discriminant validity, as well as promising indicators of its reliability. However, its criterion validity had yet to be demonstrated in an experimental context, a crucial aspect that we examine in Study 4.

### Study 4. Criterion validity of the AVAWS

In this study, we went a step further by analyzing a particular type of criterion validity, which consists of “demonstrating that the scores of a scale are related to a criterion that is predicted to be a causal antecedent of the construct that the new test is intended to measure” (Nunnally & Bernstein, 1994, p. 94). For this purpose, we used a random-group experiment to manipulate cultural (vs. personal) sexism by asking half of the participants to respond to the scale's items according to how they thought society in general would answer. We asked the other half of participants to answer according to their own opinions. This procedure stems from the experiments performed by Devine (1989) on personal vs. cultural stereotyping and from studies of the different patterns of expression of personal and societal prejudice, in which participants feel freer from normative pressures to express prejudice against groups protected by the anti-prejudice norm when the reference is what society thinks rather than what the individual thinks (Camino et al., 2001; Lima et al., 2019). This phenomenon occurs because the anti-prejudice norm has much less impact on the expression of cultural prejudice, which allows individuals to feel freer to express prejudiced judgments since they can attribute their responses to the culture instead of to themselves (Lima et al., 2019). Thus, we predicted that the participants would respond differently to the experimental manipulation of sexism (individual vs. cultural). In other words, when participants responded based on their own opinions (i.e., personal sexism), we expected them to express less acceptance of violence against women than when they responded according to what society thinks (i.e., cultural sexism). This shift in responsibility makes the individual feel freer to openly express more sexist responses (Devine, 1989), which will facilitate their support for the violence in each of the vignettes.

## Method

### Participants

A total of 300 undergraduate were randomly assigned to one of two experimental conditions: personal sexism (*N* = 150), in which they responded based on their own opinions, and cultural sexism (*N* = 150), in which they responded based on societal opinions. Most participants were female (61.7%), and their ages ranged from 18 to 63 years (M = 23.54, SD = 6.62). The majority were enrolled in humanities courses (59.3%), with psychology being the most represented field (21.8%). Participants also reported being single (85%), heterosexual (68.7%), not very religious (49.3%) and 39.4% were lower middle class. Of those surveyed, 36.5% were currently in a relationship and 24.3% had been in a relationship for more than two years. In this study, the sample size provided 80% power to detect a mean effect size of *d* = 0.50 or greater (i.e., equivalent to η^2^_p_ = 0.05), calculated using WebPower (Zhang & Yuan, 2018).

*Instruments.* We used the AVAWS scale developed in previous studies. The manipulation was performed before the presentation of each scenario (physical (α = 0.84), psychological (α = 0.86), sexual (α = 0.89), moral (α = 0.89) and economic violence (α = 0.89)), when the participants were asked to respond according to their own opinion (personal sexism condition) or society's opinion (cultural sexism condition).

*Data analysis.* The data were analyzed with IBM SPSS Statistics using ANOVA with 2 (personal sexism vs. cultural sexism) × 5 (type of violence) repeated measures, with the first factor being between-subject and the second one within-subject.

## Results

Table 4 shows the descriptive statistics for each experimental condition. The results revealed a significant main effect of the experimental manipulation, *F*(1, 297) = 156.25, *p* < 0.001, *η*^*2*^_*p*_ = 0.34 (see Table 4). As predicted, participants expressed greater support for violence against women in the cultural sexism condition (*M* = 3.09, *SE* = 0.08) than in the personal sexism condition (*M* = 1.51, *SE* = 0.09). The responses also differed according to the type of violence, *F* (4, 294) = 16.70, *p* < 0.001, *η*^*2*^_*p*_ = 0.18. We observed the following hierarchy in the support of violence: moral, economic, sexual and psychological were together in second place, and physical violence received the least support.

**Table 4:** Means and standard errors (in parentheses) of the vignettes in each experimental condition

	**Personal**	**Societal**	**Total**
Physical	1.40_b_ (.09)	2.72_d_ (.09)	2.06_d_ (.06)
Psychological	1.55_a_ (.10)	3.00_c_ (.10)	2.28_c_ (.07)
Sexual	1.47_ab_ (.10)	3.04_c_ (.10)	2.25_c_ (.07)
Moral	1.62_a_ (.10)	3.42_a_ (.10)	2.52_a_ (1.57)
Economic	1.51_ab_ (.10)	3.27_b_ (.10)	2.39_b_ (1.52)

Note. Means with distinct subscripts were significantly different in the multiple comparisons according to the least significant difference test (LSD, *p* <.05)

We also found a significant interaction between the types of violence against women and the manipulation of sexism, *F* (4, 294) = 5.37, *p* < 0.001, *η*^*2*^_*p*_ = 0.06. Multiple comparisons indicated that participants in the cultural sexism condition expressed more support for all types of violence than participants in the personal prejudice condition: physical violence (*b* = 1.32, *SE* = 0.13, *p* < 0.001, *η*^*2*^_*p*_ = 0.25), psychological violence (*b* = 1.45, *SE* = 0.14, *p* < 0.001, *η*^*2*^_*p*_ = 0.25), sexual violence (*b* = 1.58, *SE* = 0.14, *p* < 0.001, *η*^*2*^_*p*_ = 0.25), moral violence (*b* = 1.80, SE = 0.15,* p* < 0.001, *η*^*2*^_*p*_ = 0.32), and economic violence (*b* = 1.76, *SE* = 0.14, *p* < 0.001, *η*^*2*^_*p*_ = 0.33). The observed interaction showed that the differences between the experimental conditions were most pronounced in support of moral and economic violence, followed by psychological and sexual violence, with the smallest differences observed in support for physical violence. This suggests that individuals responding on the basis of personal responsibility are more likely to endorse moral and economic violence and view it as more acceptable in the context of marriage. In contrast, under the cultural sexism condition, significant differences were seen for all types of violence except sexual and psychological violence. In addition, acceptance was higher in the cultural sexism condition than in the personal sexism condition, suggesting that individuals who do not feel personally responsible are more likely to accept violence in marital relationships.

## Discussion

The present experiment extends previous studies by showing evidence for the validity of the AVAWS to capture the effects of a theoretical history of violence. That is, the AVAWS is sensitive to the manipulation of sexism: Participants showed greater acceptance of violence against women under the cultural sexism condition. In other words, respondents expressed greater societal support for violence in a situation where they were not accountable for their own judgments. A different pattern emerged when they were asked to express their own opinions. In this context of personal sexism, respondents attempted to deny that they accepted violence against women. This phenomenon has been confirmed in previous studies of other forms of prejudice (Camino et al., 2001; Devine, 1989) and of sexism-based stereotypes of competence and sociability (Fiske et al., 2002; Glick & Fiske, 1996). They showed that individuals who are strongly pressured by the anti-prejudice norm avoid expressing the prejudice individually, but they do not control their reactions when they can express their thoughts at the cultural level because they believe that society, rather than themselves, is prejudiced. It is possible that people have internalized the cultural norm of sexism and express it openly when they are allowed to, but they have distanced this norm from their attitudes. Although previous studies support this interpretation (Lima et al., 2019), it is also possible that participants expressed their cultural perception of the Brazilian context in which violence against women conforms to a descriptive norm, i.e., that there is a high rate of violence between partners in Brazil (Mapa da Violência contra a Mulher, 2018; Waiselfisz, 2015). Accordingly, it is not possible for us to know precisely to what extent the acceptance of violence under conditions of societal sexism reflects people's tendency to express their actual acceptance of violence when they are not under pressure to suppress their actual positioning, or whether they simply acknowledge that their cultural context is violent.

## General Discussion

In the four studies, we found evidence of validity and reliability of the AVAWS. In Study 1, we designed the scale and subjected it to content validity analysis. In Study 2, we explored the factorial validity of the nine vignettes that resulted from Study 1 and their respective items. Results provided consistent empirical evidence that the four items in each scenario were unidimensional, enabling us to identify the scenarios that best represented each type of violence against women. Study 3 confirmed the bifactor structure of the AVAWS by demonstrating the adequacy of the vignettes not only to evaluate each type of violence but also as a general factor independent from specific factors. Importantly, Study 3 showed evidence of the AVAWS's convergent validity with a well-established measure of violence and its discriminant validity concerning personality factors. Study 4 went one step further by experimentally testing the sensitivity of the AVAWS to capture the effect of a theoretical predictor of violence against women. Specifically, we showed that participants who were instructed to express cultural sexism scored higher than participants who were instructed to express personal sexism. Finally, in Studies 3 and 4, we observed the satisfactory reliability of the AVAWS by analyzing its internal consistency.

In sum, these results indicate sufficiently evidence that the AVAWS is a valid and reliable instrument for measuring the societal support for violence against women. In other words, the AVAWS was shown to have content validity (Study 1), factorial validity (Studies 2 and 3), convergent-discriminant validity (Study 3), and criterion validity (Study 4). Taken together, the results provide evidence of construct validity of the AVAWS. This instrument offers a new way to measure violence using actual situations from victims’ daily lives and encompassing a broad spectrum of types of violence that occur very often in victims’ homes.

### Theoretical implications

The AVAWS contributes to research on the evaluation of violence against women. Despite the existence of several other scales that assess this violence (e.g., Fincham et al., 2008; Lelaurain et al., 2018; Martín-Fernández et al., 2018; Mégias et al., 2018; Nascimento, 2015; Pimentel et al., 2017), little attention has been given to measuring violence through vignettes that portray the five types of violence in an integrated manner using typical situations. The AVAWS can significantly contribute to the study of violence because it is flexible enough to measure both generalized support for all violence against women and support for each of its specific manifestations. The results of the bifactor analysis help to understand the factor structure of the AVAWS and mainly serve to highlight the nature of the societal support for violence against women.

These results likely reflect the participants'representation of the nature of intimate relationships, especially aspects related to conflicts between partners. Indeed, both the report by the WHO (2013) and WHO (2020) about women at the international level and the Mapa da Violência Contra a Mulher [Map of Violence against Women] (2018) have already indicated that aggressors do not commit only one type of violence, but several types simultaneously or contingently. In other words, in a situation of increased tension between partners, it is possible for the male partner to deliberately attack the female economically (e.g., break her mobile phone), morally (e.g., insult her), psychologically (e.g., threaten her), sexually (e.g., force her to have sexual relations), or physically (e.g., beat her). In some situations, the male partner can commit two or more types of violence while committing only one kind in other cases. Studies 2 and 3 showed that the AVAWS is sensitive to these possibilities; it can indicate the respondents'support for the full scope of aggressive acts (i.e., expressing the G-factor) or differentiate the support for specific types of violence (i.e., S-factors). It indicates that the AVAWS can be used in different situations and contexts and is sensitive to the contingencies of each one.

One of the most significant contributions of the studies presented here is the experimental demonstration of the criterion validity of the AVAWS, which occurred in two ways. In addition to showing the sensitivity of this instrument to contextual variations that were theoretically planned to have specific effects on the instrument’s application, Study 4 went further by demonstrating, for the first time, the role played by cultural sexism on the social support for domestic violence. Indeed, the participants under the cultural sexism condition showed greater support for violence than the participants under the personal situation. These results corroborate previous studies that show how cultural prejudices can create contextual conditions for discrimination against other minority groups (e.g., Camino et al., 2001; Devine, 1989; Lima et al., 2019). As far as we know, these studies also provide the first experimental evidence of how cultural prejudice is linked to violence against women, which opens new avenues of research that can examine the possible mediators and moderators of this influence in greater detail.

The starting point is that individuals can express cultural sexism more than personal sexism. It occurs because they feel less pressured by the anti-prejudice norm and feel less responsible for expressing hostile and derogatory attitudes against the group targeted by the prejudice (e.g., Lima et al., 2019). This phenomenon makes it possible to broaden the scope of using the AVAWS to analyze the legitimizing processes of social inequalities, particularly in the context of system justification (Jost & Banaji, 1994; Jost et al., 2018), blaming the victim of domestic violence (Burt, 1980), justifying discrimination (Pereira et al., 2018), and indicating orientations toward social dominance (Pratto & Walker, 2004; Sidanius & Pratto, 1999). The AVAWS is a versatile and easy-to-use tool for experimental studies assessing individuals'motivation to legitimize violence against women. It can also be helpful in longitudinal and non-experimental studies to evaluate individual differences with possible impacts on the societal support for this violence.

### Limitations and future directions

The set of studies presented here demonstrated evidence of construct validity of the AVAWS in evaluating the societal support for domestic violence. Despite the strength and robustness of the results, the set of studies has some limitations that should be overcome in future investigations. First, the generalizability of the findings is limited due to the typical limitations of research based on university student samples. This is particularly evident in the correlational design of Studies 1 through 3, a limitation that we addressed through the experimental design of Study 4. In addition, the use of self-report and the non-representative Brazilian population in each study further limit the scope of the conclusions. In the present study, this means a less impactful concern given to the social relevance of the theme studied and the theoretical and practical implications of the AVAWS for measuring societal support for domestic violence. Future studies can use more diverse samples, including general community samples and convicted aggressors and their victims. Our study, focusing on specific samples from Brazil, necessitates a conclusion limited to this specific population. However, the potential for conducting similar studies in different countries remains strong. Conducting analogous studies in different cultural contexts may provide invaluable insights and allow the study of the psychometric parameters of the AVAWS in different societies. Such comparative analyzes would also allow for deeper exploration of the cultural influences that shape attitudes toward violence against women. By extending this research to global contexts, we can expand our understanding of societal perspectives on this critical issue and contribute to a more comprehensive and nuanced understanding of violence against women worldwide.

Another limitation concerns the underrepresented male sample. Since violence against women is mainly perpetrated by men, the underrepresentation of men could affect the results we obtained, especially in terms of the mean and variance of AVAWS. Moreover, the studies were restricted to the use of self-reported measures. Future research can consider this aspect, especially if it is possible to analyze implicit indicators of violence against women and correlate them with the AVAWS. One possible approach is to correlate the AVAWS with a measure of violence derived from the Implicit Association Test (IAT). It is important to point out that classical test theory methods were used, i.e. all items were thoroughly analyzed. In the future, an analysis based on Item Response Theory (IRT) would allow an individual assessment of each item to evaluate its discrimination and difficulty in relation to the underlying latent trait. Finally, the application of the AVAWS in longitudinal and panel studies to track indices of domestic violence would be highly beneficial as it would provide insights into the temporal stability of its psychometric properties, including test–retest reliability.

As widely discussed in modern psychometrics (Boateng et al., 2018), it is essential to emphasize that any assessment process involves some degree of measurement error variance, which includes any inherent biases. Consequently, the measurement of psychological constructs requires a systematic analysis of empirical data derived from multiple studies to progressively demonstrate validity while minimizing measurement error variance. This error variance is usually lower when the items of an instrument are embedded in a clear, well-defined and socially relevant framework (as described by Boateng et al., 2018). A highly effective way to create this frame of reference is to contextualize the items of an instrument through vignettes based on realistic social events. This approach not only helps to obtain empirical evidence of validity and reliability, but also captures the systematic variance of the constructs being measured, thus helping to minimize the variance of measurement error. The AVAWS represents a significant advance in this respect. It develops items based on well-documented theories and empirical studies (e.g., Paiva & Pereira, 2021; Pereira et al., 2018) and, more importantly, contextualizes them with socially relevant vignettes derived from news articles describing real-life situations emblematic of gender-based violence in the Brazilian context. For these reasons, the use of vignettes to contextualize scenarios of violence provides us with psychometric parameters that have fewer errors compared to instruments without such contextualization. Thus, the importance of the AVAWS for assessing support for violence is further underscored by the use of vignettes to illustrate scenarios that contextualize the psychological spaces in which violence occurs.

## Conclusions

Grounded in the theoretical framework of the Justified Discrimination Model (Pereira et al., 2018), the research program presented here employed a combination of correlational (Studies 1, 2 and 3) and experimental (Study 4) designs to systematically provide empirical evidence supporting various aspects of measurement scale validity. These include content, factorial, convergent, and discriminant, and predictive validity, which ensures that the AVAWS allow us to capture individual differences in acceptance of violence against women in a way that is both empirically valid and reliable. Despite the acknowledged limitations of our study, the results consistently support the construct validity of the AVAWS in assessing societal support for violence against women. This is particularly important in a non-WEIRD (Western, Educated, Industrialized, Rich, and Democratic) context such as Brazil, a country struggling with alarming rates of such violence. The AVAWS effectively measures both general (G-factor) and specific (S-factors) individual support for the five types of violence against women. Remarkably, this instrument is characterized by its accessibility and ease of use, making it a valuable tool in assessing an extremely concerning aspect of women's subjugation.

Violence against women is a serious social problem that directly affects their health, well-being, and socioeconomic status, and can even have deadly consequences (WHO, 2013). The AVAWS, which aims to measure societal support for this violence, can be a valuable tool for further researchers and practitioners. In addition to Brazil, where the problem is particularly acute, this tool can be invaluable for research in other Western cultures where violence against women persists despite being outlawed. By facilitating appropriate assessment of individual support for one of the most serious consequences of gender oppression—societal support for violence against women—AVAWS contributes significantly to our understanding of this deeply rooted problem. Its simplicity and effectiveness make it an indispensable tool in efforts to address and combat violence against women worldwide. It is concluded that the AVAWS is a valid and reliable instrument for assessing social support for violence against women, being a pioneer in documenting cultural sexism as an organizing principle of this support, with potential applications in clinical, educational, and legal contexts.

## Supplementary Information

Supplementary Material 1.

## Data Availability

We declare for the proper purposes that the datasets resulting from this comprehensive research program are freely available through the Open Science Foundation's repository platform (https://osf.io/5a4g3/?view_only=60a15acff6a54f0ca7c5947f99039d95).

## Abbreviations

ACVS: Acceptance of Couple Violence Scale
AMMSA: Myths about Sexual Aggression Scale
A-IPVAW: Acceptability of Intimate Partner Violence Against Women Scale
AMIVAW: Acceptance of Myths about Intimate Partner Violence Against Women
AVAWS: Acceptance of Violence Against Women Scale
CFA: Confirmatory factor analysis
CFI: Comparative Fit Index
CR: Composite Reliability
CVC: Content Validity Coefficient
CVCt: Total content validity coefficient
DVMAS: Domestic Violence Myth Acceptance Scale
EFA: Exploratory Factor Analysis
G-factor: General Violence
IAT: Implicit Association Test
ICC: Intraclass Correlation Coefficient
IRT: Item Response Theory
I-VRS: Intimate Violence Responsibility Scale
JDM: Justified Discrimination Model
RMSEA: Root Mean Square Error of Approximation
TIPI: Ten-Item Personality Inventory
TLI: Tucker-Lewis index
WEIRD: Western, Educated, Industrialized, Rich, and Democratic.
WHO: World Health Organization
WRMR: Weighted Root Mean Square Residual
