# Supplementary material for: Development and Validation of the Acceptance of Violence Against Women Scale (AVAWS)
Source: Psicol Reflex Crit. 2025 Jun 19;38:19. doi: 10.1186/s41155-025-00351-4 (PMC12179050; doi:10.1186/s41155-025-00351-4)
Supplement: Supplementary file 1 — Supplementary Material 1. [file 41155_2025_351_MOESM1_ESM.docx]

**Table S1.**

Factor loadings of the items of each scenario

| **Item** | **Scenario** | | | | | | | | |
| --- | --- | --- | --- | --- | --- | --- | --- | --- | --- |
|  | 1 | 3 | 4 | 5 | 6 | 7 | 8 | 9 | 10 |
| 1. It is understandable what _____ did. | .59 | .75 | .99 | .87 | .91 | .84 | 0.91 | .85 | .91 |
| 2._____merely fulfilled his role as a husband. | .92 | .99 | .73 | .93 | .91 | .88 | 0.90 | .89 | .86 |
| 3. _____should have kept quiet. | -.83 | -.82 | -.14 | -.74 | -.79 | -.78 | -0.59 | -.77 | -.85 |
| 4. _____’s behavior is unjustified. | -.84 | -.82 | -.17 | -.80 | -.84 | -.85 | -.85 | -.90 | -.86 |
| Eigenvalue | 2.58 | 2.88 | 1.56 | 2.80 | 2.98 | 2.81 | 2.70 | 2.91 | 3.02 |
| Explained variance (%) | 64 | 72 | 39 | 70 | 74 | 70 | 67 | 72 | 75 |
| Alpha | .84 | .85 | .56 | .88 | .90 | .88 | .87 | .91 | .92 |

Note: All of the scenarios are represented by numbers, in which 1 = physical, 3 = psychological, 4 = psychological, 5 = sexual, 6 = sexual, 7 = economic, 8 = economic, 9 = moral, 10 = moral.
